# Hydroxycinnamic acid derivatives: a potential class of natural compounds for the management of lipid metabolism and obesity

**DOI:** 10.1186/s12986-016-0080-3

**Published:** 2016-04-11

**Authors:** Md Ashraful Alam, Nusrat Subhan, Hemayet Hossain, Murad Hossain, Hasan Mahmud Reza, Md Mahbubur Rahman, M Obayed Ullah

**Affiliations:** Department of Pharmaceutical Sciences, North South University Bangladesh, Dhaka, Bangladesh; School of Biomedical Sciences, Charles Sturt University, Wagga Wagga, New South Wales Australia; BCSIR Laboratories, Bangladesh Council of Scientific and Industrial Research (BCSIR), Dhaka, Bangladesh

**Keywords:** Hydroxycinnamic acid, Obesity, Diabetes, Dyslipidemia, Inflammation

## Abstract

Hydroxycinnamic acid derivatives are important class of polyphenolic compounds originated from the Mavolanate-Shikimate biosynthesis pathways in plants. Several simple phenolic compounds such as cinnamic acid, *p*-coumaric acid, ferulic acid, caffeic acid, chlorgenic acid, and rosmarinic acid belong to this class. These phenolic compounds possess potent antioxidant and anti-inflammatory properties. These compounds were also showed potential therapeutic benefit in experimental diabetes and hyperlipidemia. Recent evidences also suggest that they may serve as valuable molecule for the treatment of obesity related health complications. In adipose tissues, hydroxycinnamic acid derivatives inhibit macrophage infiltration and nuclear factor κB (NF-κB) activation in obese animals. Hydroxycinnamic acid derivatives also reduce the expression of the potent proinflammatory adipokines tumor necrosis factor-α (TNFα), monocyte chemoattractant protein-1 (MCP-1), and plasminogen activator inhibitor type-1 (PAI-1), and they increase the secretion of an anti-inflammatory agent adiponectin from adipocytes. Furthermore, hydroxycinnamic acid derivatives also prevent adipocyte differentiation and lower lipid profile in experimental animals. Through these diverse mechanisms hydroxycinnamic acid derivatives reduce obesity and curtail associated adverse health complications.

## Background

Metabolic syndrome is a cluster of non-communicable diseases includes central obesity, diabetes, insulin resistance, hypertension and dyslipidemia. Prevalence of obesity and diabetes are increasing day by day among children, young and elderly populations both in developed and developing countries [1–3]. A sedentary nature of jobs and high calorie diet mainly western style diet are the main causes of developing obesity and diabetes, consequently metabolic syndrome [1, 4]. Ever increasing obese and diabetes population are causing serious problems for the management of health sector as well as increasing personal health risks [4]. Recent evidence also suggests that increased body fat mass causes cardiovascular diseases and increases morbidity and mortality in human [5, 6]. Dietary modification, for example decreasing the intake of high fat and high carbohydrate could be a possible way of reducing the risk of fat accumulation in the body. In addition, several dietary approaches such as Mediterranean diet or diet containing high amount of fibres, fruits and vegetables would be valuable for the prevention of hypertension, diabetes, dyslipidemia and obesity [7, 8]. Mediterranean diet or fruits and vegetables possess large amount of phenolic or polyphenolic compounds. It is now widely recognized that, phenolic or polyphenolic compounds are strong antioxidant substances and showed anti-inflammatory properties [9, 10]. Some of them are also effective against diabetes, insulin resistance and dyslipidemia [11–15]. Many of them also prevent hypertension and cardiovascular diseases [16]. All these biological activities are mainly regulated by phenolic acid’s ability to scavenge free radicals generated due to excess nutrition supply to the tissues in obesity, or they may regulate the energy homeostasis and inflammatory pathways. This work will thus review the potential health benefit of hydroxycinnamic acid derivatives in obesity and metabolic syndrome and their possible mechanism of action.

### Obesity and energy homeostasis, mechanism of fat metabolism

Obesity can be defined as the accumulation of excess fat due to the increased energy intake and lack of energy expenditure. However, World Health Organization uses Body Mass Index (BMI) as a parameter for defining obesity. According to WHO, BMI >30 is considered as moderately obese and BMI > 35 is considered as severely obese in human [17]. Global obesity in young to adult population is increasing tremendously in recent years [18, 19]. Lack of physical movements, sedentary nature of work and consumption of diet containing high carbohydrate and high fat are responsible for the development of obesity [20]. Thus, increased energy expenditure would be a contributing factor to control and manage obesity and related pathophysiological conditions. Mitochondrial biogenesis are the major pathways in various cell types like, liver, adipose tissue, skeletal muscle etc. to increase ATP production and energy expenditure. Decreased mitochondrial function was observed in obesity and metabolic disorder [21–23]. In obese condition, abundance of fuel supply e.g., fatty acid and glucose overwhelm the mitochondrial electron transport chain and increased the superoxide production [24–27]. Mitochondrial biogeneses are regulated via several transcriptional regulatory factors like AMPK, PPAR- γ and PGC-1α [28, 29]. AMPK regulated PPAR-γ and PGC-1α activation stimulated most of the transcriptional signal to increase fatty acid oxidation and mitochondrial function [30–32].

#### AMPK

AMP-activated protein kinase (AMPK) is a cellular fuel gauge, maintaining intracellular energy balance in mammalian cells [33]. AMPK signalling pathway is activated by elevation of the AMP/ATP ratio due to the decreased ATP synthesis by mitochondria or by increased energy (ATP) expenditure [33]. Glucose deprivation, hypoxia or ischaemia, or metabolic poisons are few factors which may inhibit glycolysis, tricarboxylic acid cycle or oxidative phosphorylation and disturb energy balance by interfering with ATP synthesis, which may trigger activation of AMPK signalling [34]. AMPK activation is necessary for the transcriptional regulation of energy demand. Mice expressing a dominant-negative form of AMPK failed to increase mitochondrial biogenesis in response to energy deprivation in skeletal muscles [35]. In contrast, lipid oxidation and mitochondrial activity was increased in mice over expressing the phosphorylated AMPK [36, 37]. Several ligands such as thiazolidinediones (for example, rosiglitazone) and biguanides (metformin) both activates AMPK [38]. Thiazolidinediones and biguanides inhibits complex I of the mitochondrial respiratory chain and elevates cellular AMP/ATP ratios [39]. Furthermore, mice fed with AMPK agonists increased oxidative gene expression, enhanced endurance capacity and gave protection against metabolic disease [40, 41]. AMPK can also be activated by metabolic stresses such as muscle contraction or hypoxia, and modulated by hormones and cytokines affecting whole-body energy balance such as leptin, adiponectin, resistin, ghrelin and cannabinoids [33].

#### PPAR- γ

AMPK activation increased the fatty acid oxidation through activating the PPAR-γ and PGC-1α [42]. Peroxisome proliferator activator protein-γ (PPAR- γ) is highly expressed in adipose tissues [43]. The expression of PPARγ in liver is very low compared to the level present in adipose tissue [43–45]. The actions of PPAR-γ are mediated by two protein isoforms, PPARγ1 and PPARγ2 [46]. PPARγ1 is widely expressed while PPARγ2 is restricted to the adipose tissue only [46]. Fatty acids binding activates PPAR-γ [46]. Activation of PPARγ is necessary for adipocyte differentiation and fatty-acid storage [43, 44]. PPAR-γ deficient mice are devoid of adipose tissue and PPAR-γ +/− mice are characterized by a decreased adipose tissue mass [47, 48]. PPAR-γ is also important for anti-inflammatory pathways, lipid metabolism and regulates genes taking part in the release, transport and storage of fatty acids [49, 50]. Moreover, PPARγ is also responsible for the improvement of insulin resistance and plays an important role in glucose homeostasis. Mice lacking PPARγ in fat, muscle, or liver are predisposed to develop insulin resistance [51–54] while mice with increased PPARγ activities are protected from obesity-associated insulin resistance [55]. PPARγ is a ligand activated protein, thiazolidinediones are considered as the activator of PPARγ [56]. However, thiazolidinediones are adipogenic and responsible for moderate weight gain in patients taking thiazolidinediones [50, 57].

#### PGC-1α

Peroxisome proliferator activator protein-γ co-activator-1α (PGC-1α) is another regulator of lipid and glucose metabolism. AMPK regulates PGC-1α at both gene and protein level [36]. PGC-1α directly co-activates multiple transcriptional factors such as the PPARs or the thyroid hormone receptor, glucocorticoid receptors and estrogen receptors [29, 58]. PGC-1α also increases mitochondrial biogenesis and respiration rates, as well as the uptake and utilization of substrates for energy production [59]. In brown adipose tissue (BAT), cold induces PGC-1α protein expression that controls adaptive thermogenesis [59]. Furthermore, fasting induces hepatic PGC-1α expression and increases gluconeogenesis, whereas in skeletal and cardiac muscle, exercise increases PGC-1α mediated mitochondrial biogenesis and respiration [60]. Phenolic compound resveratrol increased the PGC-1α activity and increased running time and consumption of oxygen in muscle fibers in mice [61]. Moreover, resveratrol increased insulin sensitivity, reduced insulin-like growth factor-1 (IGF-I) levels, increased AMP-activated protein kinase (AMPK) and peroxisome proliferator-activated receptor-gamma co-activator-1α (PGC-1α) activity, increased mitochondrial number, and improved motor function in middle-aged mice fed a high-calorie diet [62].

## Inflammation and obesity

Inflammation is a protective response mechanism for tissue injury. Both acute and chronic inflammatory responses are responsible for the development of diabetes and insulin resistance [63, 64]. Recent research findings suggest that chronic low grade inflammation is developed in obese individuals and triggers adipocyte dysfunction [65]. Moreover, adipose tissues are playing a major role in secreting pro-inflammatory and inflammatory cytokines during obesity [66]. Pathologic growth of adipocyte houses many of inflammatory cytokines like TNF-alpha and IL-6 [67]. Inputs into this inflammatory response further stimulate ER stress, adipose tissue hypoxia, and adipocyte death [68–70]. Macrophage numbers in adipose tissues are also increased with obesity where they mainly scavenge the dead adipocytes [71, 72]. Macrophages are also responsible for the cytokine production in obese adipose tissues [73].

## Hydroxycinnamic acid derivatives overview

**Fig. 1:**
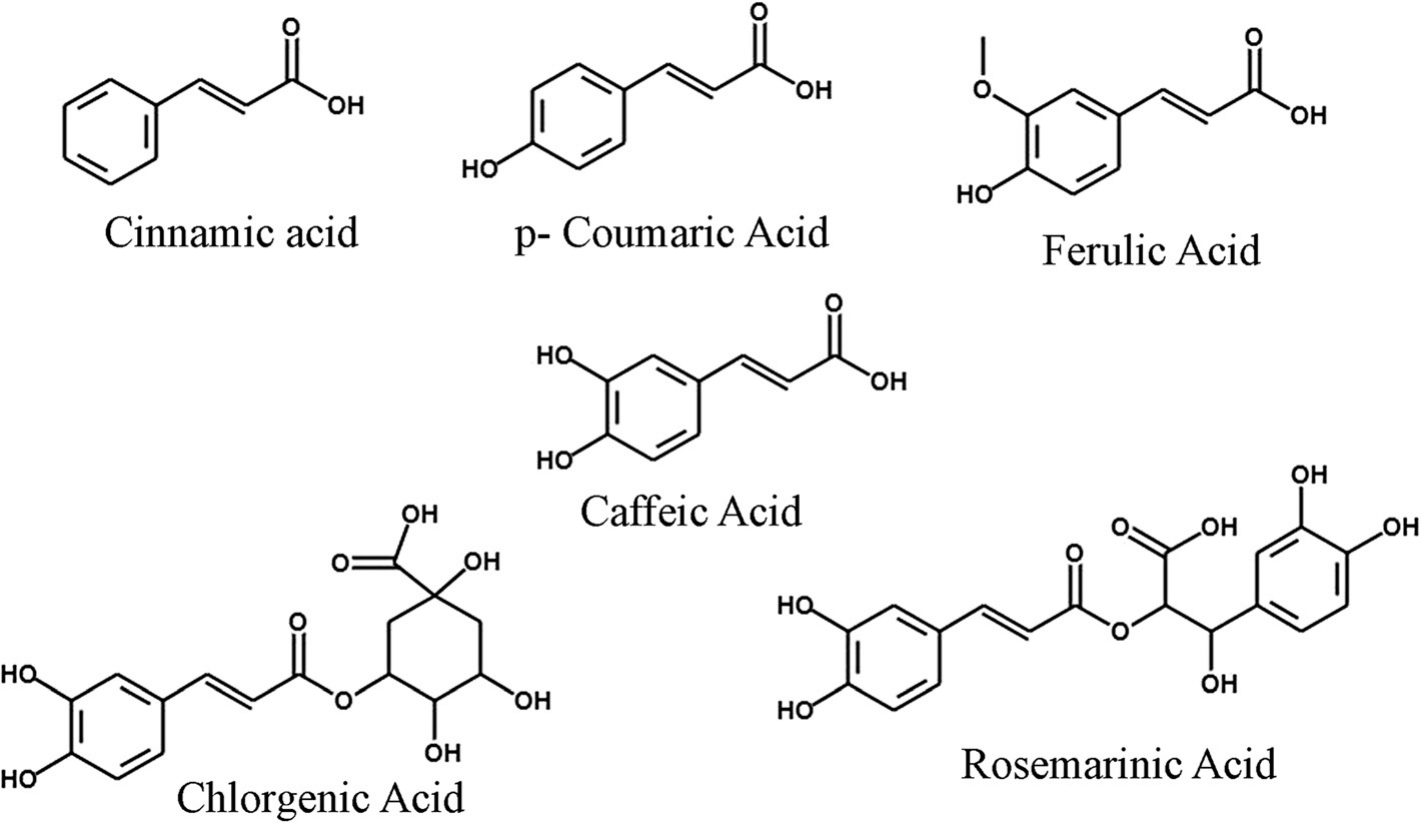
Structures of hydroxyl cinnamic acid derivatives. Cinnamic acid, *p-*caumaric acid, ferulic acid, caffeic acid, chlorgenic acid, rosemarinic acid

Hydroxycinnamic acid derivatives (Fig. 1) comprise a large group of simple phenolic acids, found mainly in cereals, fruits and vegetables. A review has been published recently describing the occurrence, biosynthesis, and pharmacokinetics of hydroxycinnamic acid derivatives [74]. Ferulic acid, caffeic acid, *p-*coumaric acid, chlorgenic acid, sinapic acid, curcumin, and rosmarinic acid belongs to this important phenolic acid group. Hydroxycinnamic acids are abundant in fruits, vegetables and cereals and seeds of fruits [74]. In plant, hydroxycinnamic acid derivatives are synthesized by following the mavolonate and shikimate pathways where phenylalanine and tyrosine are two starter precursor molecules [74, 75]. Following several intermediate enzymatic process, shikimate pathways produced cinnamic acid, *p*-coumaric acid, caffeic acid, ferulic acid, sinapic acid to various complex lignin molecules [74, 75]. Hydroxycinnamic acid derivatives are also serving as precursor molecules for the stilbenes, chalcons, flavonoids, lignans, and anthocyanins [74].

**Fig. 2:**
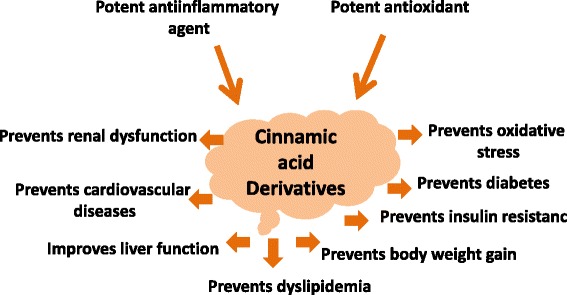
Health benefit of cinnamic acid derivatives in various diseases

HCAs are absorbed easily from the stomach and intestine depending on their structure and compared to other complex phenolic compounds [76]. Ferulic acid and *p*-coumaric acid are also absorbed from intestine, jejunum, ileum and colon of rats [74]. However, chlorgenic acid, ester of caffeic and quinic acid, first hydrolysed and free caffeic acid is absorbed from the intestine [76]. In Caco-2 cell monolayers, caffeic acid demonstrated that monocarboxylic acid transporters (MCTs), a transport system present across the intestinal epithelial cells, may be involved in the absorption process [77, 78]. *p*-Coumaric acid and ferulic acid also followed the same monocarboxylic acid transporter (MCT) system to cross the intestinal epithelium [77, 78]. However, passive diffusion mechanism is also important and not ignored for the absorption of ferulic acid in the stomach and Caco-2 cells [75, 79]. In addition, involvement of a Na^+^-dependent, carrier-mediated transport process are also involved in the uptake of cinnamic acid and ferulic acid across the brush border membrane of rat jejunum [80]. Bioavailability of cinnamic acid derivatives are reviewed recently [74, 76]. Various cinnamic acid derivatives can be found in plasma immediately after the oral administration and may show various health benefit in different diseases (Fig. 2).

## Effect of Hydroxycinnamic acid derivatives on various parameters of Metabolic syndrome

### Effect of hydroxycinnamic acid derivatives in Inflammation

Hydroxycinnamic acid derivatives showed anti-inflammatory properties both in vitro and in vivo [81]. Ferulic acid prevented the production of TNF-alpha and decreased Macrophage inflammatory protein-2 (MIP-2) levels in lipopolysaccharide (LPS)-stimulated RAW264.7 cells [82]. The transcription factor nuclear factor kappa B (NF-κB) plays a critical role in stress, immune, and inflammatory responses. Ferulic acid in cereals inhibits NF-kB activation [83]. Salt of ferulic acid, ferulate, exhibited antioxidant action by maintaining redox regulation, suppressing NF-κB activation and modulating the expression of NF-κB-induced, proinflammatory COX-2, iNOS, VCAM-1 and ICAM-1 in aged Sprague–Dawley rats [84]. NF-kB suppression by ferulate is mediated via suppressing the activation of NIK/IKK and MAPKs [84].

*p*-Coumaric acid prevented the increased cell-mediated immune responses and macrophage phagocytic index in rats [85].*p*- Coumaric acid also decrease in the expression of inflammatory mediator TNF-α and circulating immune complexes in adjuvant induced arthritic rats [85]. *p*-Coumaric acid also inhibited the TNF-α-induced changes in levels of monocyte chemoattractant protein-1 (MCP-1), plasminogen activator inhibitor-1 (PAI-1), and intracellular reactive oxygen species (ROS) in 3 T3-L1 adipocytes [86]. Furthermore, *p-*coumaric acid increased the secretion and concentration of adiponectin, superoxide dismutase (SOD), glutathione (GSH), glutathione peroxidase (GPx), and glutathione S-transferase (GST) in TNF-α-treated 3 T3-L1 adipocytes [86].

Caffeic acid phenetyl ester (CAPE) non-selectively inhibited the activities of baculovirus-expressed hCOX-1 and hCOX-2 enzymes and inhibits prostaglandin synthesis and COX 2 in the rat carrageenan air pouch model of inflammation [87]. Caffeic acid and some of its derivatives such as caffeic acid phenetyl ester (CAPE) and octyl caffeate showed anti-inflammatory activity both in vitro and in vivo [88]. Caffeic acid derivatives suppressed the iNOS expression and prevented the production of NO from RAW macrophage cells. Moreover, butyl, octyl and CAPE derivatives of caffeic acid inhibited carrageenan-induced paw edema and prevented the increase in IL-1β levels in the mouse paw [88]. Butyl, octyl and CAPE derivatives also prevented carrageenan-induced neutrophil influx in the mouse paw [88]. Caffeic acid supplementation reduced the inflammatory cytokines interleukin (IL)-beta, IL-6, tumor necrosis factor (TNF)-alpha and monocyte chemoattractant protein (MCP)-1 concentration in diabetic mice [16].

Chlorogenic acid, the ester of caffeic acid with quinic acid blocked UVB- or TPA-induced transactivation of AP-1 and NF-κB in JB6 P^+^ cells [89]. CGA inhibited lipopolysaccharide (LPS)-induced inflammatory response in RAW 264.7 cells mediated by decreasing cyclooxygenase (COX-2) at protein and mRNA levels and decreased the secretion of prostaglandin E2 (PGE2) [90]. Chlorogenic acid also inhibited LPS induced inflammation of liver in mice and prevented the mRNA expression of toll-like receptor 4 (TLR4), TNF-α and NF-κB p65 subunit [91].

### Effect of hydroxycinnamic acid derivatives on lipid and fat metabolism

Elevated plasma concentrations of total cholesterol (TC) and low density lipoprotein (LDL) cholesterol (and/or reduced high-density lipoprotein [HDL]) are commonly seen in dyslipidemia and strongly associated with cardiovascular disease, peripheral vascular disease and stroke [92]. High fat diet feeding in laboratory animals showed dyslipidemic condition similar to human dyslipidemia. Several plant based compounds e.g., plant stanols and sterols, tea-based catechins and theaflavins showed improvement in lowering plasma lipid profiles; however the clinical efficacy of many of these substances are not well studied [93]. Most of the hydroxycinnamic acid derivatives are effective against fat deposition and lowered plasma lipid profile and increases fat metabolism in liver (Table 1). Polyphenol rich red wine improved plasma lipid profiles by increasing HDL cholesterol levels, improve LDL oxidation [94] and improved the antioxidant status by reducing the oxidative stress in patients [95].

**Table 1:** Lipid lowering effect of hydroxycinnamic acid derivatives

Derivatives	Model	Experimental outcome	Reference
Cinnamic acid	High Cholesterol fed rats (Cinnamic acid (0.02 %, w/w)	- Inhibited hepatic HMG-CoA reductase and ACAT activity. - Reduced the elevated AST and AST concentration in plasma. - Lowered plasma and liver triglycerides and cholesterol concentrations. - Improved antioxidant ezymes activities in erythrocytes and liver.	[96]
	Cinnamic acid (30 mg/kg/day) for 7 weeks	- The administration of CA to HFD-fed rats reduced the body weight gain - Reduced serum lipid profile and - Reverted back near to normal of lipase and ACE enzymes activities	[122]
	HFD diet fed Male Wistar rats		
Ferulic acid	C57BL/6 mice fed with high fat diet.	- Lowered liver and plasma cholesterol by reducing fatty acid synthase and glucose 6 phosphate dehydrogenase	[97]
	Golden syrian hamsters (chow-based hypercholesterolemic diet (HCD) containing 10 % coconut oil and 0.1 % cholesterol for 2 weeks)	- Lowered plasma plasma lipid and lipoprotein cholesterol concentrations. - Preserved the antioxidant status by preserving higher amount of Vitamin E in plasma.	[98]
	Stroke-prone spontaneously hypertensive rats (SHRSP)	- Plasma total cholesterol and triglyceride levels were lower after 2 h administration. - The mRNA expression of genes involved in lipid and drug metabolism was downregulated	[99]
	Apolipoprotein E-deficient (apo E−/−) mice fed Western	- Lowered the Concentrations of total cholesterol (total-C), apolipoprotein B (apo B) in the plasma and epididymal adipose tissue wet weight - Lowered hepatic ACAT and HMG-CoA reductase were only significantly.	[101]
	Male apo E−/− mice	- Lowered The hepatic and erythrocyte thiobarbituric acid-reactive substances levels. - Lowered the plasma total cholesterol concentration accompanied with a decreased hepatic acyl-coenzyme A: cholesterol acyltransferase activity.	[100]
	Streptozotocin induced diabetes rat	- Reduced the elevated plasma lipid and blood glucose levels	[102]
Caffeic acid	High fat diet in mice (30 mg/kg of CAPE)	- Reduced plasma cholesterol and triglycerides. - Amelioration in hepatic steatosis. - Increase in glucose sensitivity by improving phosphorylation of the insulin receptor substrate-2 and Akt phosphorylation. - Reduced the induction of the inflammatory pathway, c-jun-N- terminal kinase, the nuclear factor kappa B, and cyclooxygenase-2 expression.	[117]
Chlorogenic acid	ICR mice fed with high fat diet.	- Lowered plasma cholesterol by reducing the activity of fatty acid synthase and HMG-CoA reductase and increased the fatty acid beta oxidation.	[15]
	Fa/fa Zucker Rats	- Lowered plasma fasting cholesterol and triglycerides	[103]
	Streptozotocin (STZ)–nicotinamide (NA)-induced type 2 diabetic rats. (CGA 5 mg/kg)	- Lowered the plasma lipid; cholesterol, free faty acids and triglycerides. - Lowered HMG-CoA reductase activity in liver and increased LPL activity in plasma.	[104]

Cinnamic acid derivative supplementation lowered the plasma and liver triglycerides and cholesterol concentrations in high cholesterol fed rats [96]. Ferulic acid supplementation also lowered plasma lipid and cholesterol concentrations in various model of dyslipidemia such as C57BL/6 mice fed with high fat diet [97], Golden syrian hamsters fed with chow-based hypercholesterolemic diet [98], stroke-prone spontaneously hypertensive rats [99], apolipoprotein E-deficient (apo E−/−) mice fed Western diet [100, 101] and in streptozotocin induced diabetes rats [102]. Chlorogenic acid infusion in diabetic Zucker rats lowered the fasting plasma cholesterol and triacylglycerol concentrations significantly [103]. Chlorogenic acid also lowered the plasma cholesterol in ICR mice fed with high fat diet [15] and lipid, free fatty acids and triglycerides in Streptozotocin (STZ)–nicotinamide (NA)-induced type 2 diabetic rats [104].

Cholesterol lowering effect is attributed to the inhibition of the cholesterol synthesis and utilization of the free fatty acids in liver. HMG-CoA reductase is the rate regulating enzymes found in liver which is responsible for the cholesterol biosynthesis. Several statins selectively inhibited the HMG-CoA reductase in liver and lowered plasma cholesterol in hyperlipidemic patients [105, 106]. Hepatic ACAT is other type of enzymes that increased the utilization of fatty acid for cholesterol biosynthesis. Cinnamic acid derivatives such as cinnamic acid, ferulic acid, chlorgenic acid reduced the HMG-CoA reductase and ACAT activity in experimental animals [96, 101, 15, 104]. Ferulic acid decreased hepatic acyl-coenzyme A: cholesterol acyltransferase activity [100, 101] and down regulates the genes involved in lipid metabolism [99]. Moreover, Chlorgenic acid increased beta-oxidation and lypolitic lipase activity in diabetic animal [15, 104].

### Effect of hydroxycinnamic acid derivatives on body weight and obesity

Hydroxycinnamic acids are also effective against body weight gain, fat deposition and dysfunction of the adipocytes due to high fat diet feeding in animal model (Table 2). Adipocyte proliferation and differentiation plays critical role on adipose tissue deposition and dysfunction. 3 T3-L1 preadipocytes are excellent cell lines for studying the anti-obesity effect of various therapeutic agents. Addition of phenolic acids to the growth medium decreased the cell population of 3 T3-L1 preadipocytes in vitro [107]. Chlorogenic acid, *o*-coumaric acid, and *m*-coumaric acid caused cell cycle arrest in the G1 phase in 3 T3-L1 preadipocytes [107]. Ferulic acid prevented the body weight gain in high fat diet fed mice and decreased the plasma and liver lipids, triglycerides and total cholesterol [97]. Ferulic acid also decreased the activity of hepatic lipogenic enzymes, such as G6PD, ME, and FAS which are responsible for the cholesterol and fatty acid synthesis [97]. Chlorogenic acid showed anti-obesity effect on mice fed with a high fat diet [15]. Chlorogenic acid also lowered the visceral fat mass and plasma leptin and insulin levels compared to the high-fat control group [15]. Caffeic acid and chlorogenic acid significantly inhibited fatty acid synthase, 3-hydroxy-3-methylglutaryl CoA reductase and acyl-CoA:cholesterol acyltransferase activities, while they increased fatty acid β-oxidation activity and peroxisome proliferator-activated receptors α expression in the liver compared to the high-fat group [15].

**Table 2:** Effect of hydroxycinnamic acid derivatives on obesity and adipocyte dysfunction

Derivatives	Model	Experimental outcome	Reference
Cinnamic acid	3 T3-L1 adipocytes	- Stimulated the secretion of adiponectin and the phosphorylation of AMPK in 3 T3-L1 adipocytes and therefore improves insulin sensitivity	[123]
	- Cinnamic acid (30 mg/kg/day) for 7 weeks	- The administration of CA to HFD-fed rats reduced the body weight gain.	[122]
	- HFD diet fed Male Wistar rats		
Coumaric acid	3 T3-L1 adipocytes	- Inhibition of adipogenesis in 3 T3-L1 adipocytes.	[124]
		- o-coumaric acid inhibited GPDH activity and the expression of PPARγ, C/EBPα and leptin and then up-regulated expression of adiponectin.	
	3 T3-L1 adipocytes	- p-Coumaric acid inhibited TNF-α-induced changes in levels of monocyte chemoattractant protein-1 (MCP-1), plasminogen activator inhibitor-1 (PAI-1), and intracellular reactive oxygen species (ROS) in 3 T3-L1 adipocytes.	[125]
		- p-Coumaric acid increased the secretion of adiponectin, superoxide dismutase (SOD), glutathione (GSH), glutathione peroxidase (GPx), and glutathione S-transferase (GST) in TNF-α-treated 3 T3-L1 adipocytes	
	Wistar rats fed a high fat diet.(100 mg/kg)	- Decreased body weight, liver organ, and adipose tissue weights of peritoneal and epididymal fat pads.	[12]
		- Decreased hepatic triacylglycerol and cholesterol levels.	
		- Enhanced the levels of glutathione (GSH), GSH peroxidase (GPx), GSH reductase (GRd), and GSH S-transferase (GST) in the hepatic tissue	
Ferulic acid	high fat diet-induced obesity in mice	- Oryzanol or ferulic acid significantly suppressed the weight gain of the high fat diet-induced obesity in mice.	[97]
		- Ferulic acid is more effectively suppressed the weight gain compared to oryzanol.	
Caffeic acid	3 T3-L1 adipocytes	- Inhibitory effects on increased glycerol-3-phosphate dehydrogenase (GPDH) activity and an increased insulin receptor substrate 1 (IRS-1).	[126]
		- Reduced the levels of leptin, resistin, and tumor necrosis factor (TNF)-alpha.	
	High-fat diet induced obese mice (0.02 % CFA of diet (wt/wt) dose)	- Lowered body weight, visceral fat mass and plasma leptin and insulin levels.	[15]
		- Inhibited fatty acid synthase, 3-hydroxy-3-methylglutaryl CoA reductase and acyl-CoA:cholesterol acyltransferase activities.	
		- increased fatty acid β-oxidation activity and peroxisome proliferator-activated receptors α expression in the liver	
Chlorogenic acid	High-fat diet induced obese mice (0.02 % CGA of diet (wt/wt) dose)	- Lowered body weight, visceral fat mass and plasma leptin and insulin levels.	[15]
		- Inhibited fatty acidsynthase, 3-hydroxy-3-methylglutaryl CoA reductase and acyl-CoA:cholesterol acyltransferase activities.	
		- Increased fatty acid β-oxidation activity and peroxisome proliferator-activated receptors α expression in the liver.	
	Streptozotocin (STZ)–nicotinamide (NA)-induced type 2 diabetic rats CGA (5 mg/kg b.w.)	- Decreased plasma and tissue triglycerides, free fatty acids.	[104]
		- Decreased the activity of HMG-CoA reductase.	
		- Prevents lipid accumulation in liver.	
	Insulin resistant *(fa/fa)* Zucker rats (infused CGA 5 mg/Kg body weight/day)	- Fasting plasma cholesterol and triacylglycerols concentrations were significantly decreased.	[103]
	Golden hamsters (80 mg CGA/kg body weight daily given peritonially)	- Lowered fasting serum triglyceride (TG), free fatty acid (FFA), total cholesterol (TC), low density lipoprotein cholesterol (LDL-C), high density lipoprotein cholesterol (HDL-C), glucose (FSG), and insulin (FSI).	[127]
		- Increased hepatic lipase (HL), lower contents of TG and FFA in liver and lower activity of lipoprotein lipase (LPL) in skeletal muscle.	
		- Elevated the expression level of mRNA and protein expression in hepatic PPAR-α.	
	High Cholesterol diet fed Sprague–Dawley rats (1 or 10 mg/kg/day p.o. CGA)	- Lowered total cholesterol, triglycerides, high-density lipoprotein and low-density lipoprotein.	[128]
		- Up-regulated of peroxisome proliferation-activated receptor α mRNA in liver.	

### Effect of hydroxycinnamic acid derivatives on diabetes and insulin resistance

Hyperglycemia and insulin resistance are commonly seen in obesity [108–110]. Polyphenolic compounds showed prevention of metabolic disorder associated with hyperglycemia and diabetes. The mechanisms behind these benefits have multiple targets. Some molecules prevented the beta cell destruction in pancreas thereby increasing the insulin secretion. Others include inhibition of carbohydrate digestive enzymes, increased glycogen synthesis, increased glucose uptake in muscle tissues and adipocytes by phosphorylation of AMPK and increased GLUT4 content as well as increasing glucose metabolism. Hydroxycinnamic acid derivatives also showed considerable hypoglycaemic activities in experimental condition (Table 3). Cinnamic acid improved glucose intolerance and insulin resistance in STZ induced diabetic rats [13]. Cinnamic acid also increased the expression of glycogen synthase, whereas the expression of glycogen synthase kinase and phosphorylation of glycogen synthase at Ser641 in TNF-α-treated insulin-resistant mouse hepatocytes was decreased [111]. Rice bran fraction and ferulic acid reduced the blood glucose concentrations and increased the insulin in plasma of diabetic C57BL/KsJ db/db mice [112]. Glucose lowering effect by ferulic acid was also seen in KK-A^y^ mice [14] and STZ induced diabetic mice [14]. Ferulic acid also increased glucokinase activity [112] and decreased glucose-6-phosphatase (G6pase) and phosphoenolpyruvate carboxykinase (PEPCK) activities in liver [113]. Moreover, ferulic acid prevented lipid peroxidation and improved the antioxidant enzymes such as glutathione peroxidase (GPx), superoxide dismutase (SOD) and catalase (CAT) [114].

**Table 3:** Effect of hydroxycinnamic acid derivatives on diabetes

Derivatives	Model	Experimental outcome	Reference
Cinnamic acid	TNF-α-treated insulin-resistant mouse FL83B hepatocytes.	- Increased expression of glycogen synthase, whereas the expression of glycogen synthase kinase and phosphorylation of glycogen synthase at Ser641 in insulin-resistant mouse hepatocytes was decreased.	[111]
	STZ-induced diabetic Wistar Albino rats	- Improved glucose tolerance and carbohydrate metabolizing enzymes,	[13]
Ferulic acid	STZ-induced diabetic mice (0.01 and 0.1 % FA of diet)	- Decreased elevated blood glucose level	[14]
	KK-A^y^ mice (0.05 % FA of Diet)	- Suppress blood glucose level	[14]
	C57BL/KsJ db/db mice	- Decreased blood glucose level by increasing glycogen synthesis. Increased glucokinase activity.	[112]
	Streptozotocin induced diabetes rats	- Prevents lipid peroxidation and improved the antioxidant enzymes such as glutathione peroxidase (GPx), superoxide dismutase (SOD) and catalase (CAT)	[114]
	Otsuka Long-Evans Tokushima Fatty (OLETF) diabetic rats (0.2 % FA in diet)	- Improved	
	Male C57BL/6 N mice (0.5 % FA of diet)	- Lower blood glucose level and glucose-6-phosphatase (G6pase) and phosphoenolpyruvate carboxykinase (PEPCK) activities.	[113]
	Stroke-prone spontaneously hypertensive rats (SHRsp) (0 · 01 g/kg FA of diet)	- Improved hypertension as well as glucose tolerance, plasma nitric oxide (NOx). Also increased several mRNA expressions of metabolic parameters involved in glucose and lipid metabolisms	[129]
	- high-fat and fructose-induced type 2 diabetic adult male rats	- FA treatment to diabetic animals restored blood glucose, serum insulin, glucose tolerance, and insulin tolerance to normal range. - Hepatic glycogen concentration, activity of glycogen synthase, and glucokinase were significantly increased, whereas activity of glycogen phosphorylase and enzymes of gluconeogenesis (phosphoenolpyruvate carboxykinase (PEPCK) and glucose-6-phosphatase (G6Pase)) were decreased in diabetic animals due to FA treatment	[130]
	- FA (50 mg/(kg body weight · day)(−1), orally) for 30 days		
	- high-fat and fructose-induced type 2 diabetic adult male rats	- Authors suggested that FA treatment reduced the GLUT2 expression in diabetic animals by impairing the interaction between these transcription factors (SREBP1c, HNF1α and HNF3β) and GLUT2 gene promoter.	[131]
	- FA (50 mg/(kg body weight · day)(−1), orally) for 30 days		
Caffeic acid	C57BL/KsJ-*db/db* mice	- Reduction of the blood glucose and glycosylated hemoglobin levels. Caffeic acid also markedly increased glucokinase activity and its mRNA expression and glycogen content and simultaneously lowered glucose-6-phosphatase and phosphoenolpyruvate carboxykinase activities and their respective mRNA expressions, accompanied by a reduction in the glucose transporter 2 expression in the liver	[115]
	Streptozotocin induced diabetes rats	- Improved lipid peroxidation and antioxidant enzyme status in liver of rats	[132]
	Mouse liver FL83B cells	- Tumor necrosis factor-α was used to induce insulin resistance. may promote insulin receptor tyrosyl phosphorylation, up-regulate the expression of insulin signal associated proteins, including insulin receptor, phosphatidylinositol-3 kinase, glycogen synthase, and glucose transporter-2, increase the uptake of glucose, and alleviate insulin resistance	[118]
	TNF-α-treated insulin-resistant mouse FL83B hepatocytes.	- Increased expression of glycogen synthase, whereas the expression of glycogen synthase kinase and phosphorylation of glycogen synthase at Ser641 in insulin-resistant mouse hepatocytes was decreased. - Also suppressed the expression of hepatic nuclear factor-4 and activity of phosphoenolpyruvate carboxykinase	[111]
	High fat diet in male BLTW: CD1(ICR) mice	- Improved the glucose intolearance and normalized plasma insulin, adiponectin. - also suppress TNF-alpha, PEPCK and increased GLUT4	[119]
	L6-GLUT4*myc* cells	- Increased glucose uptake and GLUT4 translocation to the cell membrane of L6-GLUT4myc cells. - Increased phosphorylation of AMPK and increased GLUT4 content	[120]
	Streptozotocin (STZ)-induced diabetic rats	- Phoshoenolpyruvate carboxykinase mRNA expression was decreased. - Decreased the fasting blood levels of glucose, alanine aminotransferase, cholesterol, and triglyceride induced by diabetes. - increased expressions of glucokinase and pyruvate kinase mRNAs and increased the liver glycogen level.	[116]
	Swiss mice fed high fat diet	- Improved glucose intolerance in high fat diet fed mice. - Improvement in insulin-stimulated phosphorylation of the insulin receptor substrate-2, followed by an increase in Akt phosphorylation. - Reduced the induction of the inflammatory pathway, c-jun-N- terminal kinase, the nuclear factor kappa B, and cyclooxygenase-2 expression.	[117]
	Male Sprague–Dawley rats	- Increased the phosphorylation of AMPKα Thr172 in skeletal muscle. - AMPKα2 activity increased significantly, whereas AMPKα1 activity did not change.	[133]
	Male Balb/cA mice (2.5 % CFA of Diet)	- Increased plasma insulin and decreased blood glucose and plasma HbA1c levels. - Lowered renal levels of IL-6, IL-1b, tumor necrosis factor (TNF)-a and monocyte chemoattractant protein 1 (MCP-1) and decreased TNF alpha and MCP-1 mRNA expression.	[11]
Chlorogenic acid	db/db mice	- Improved the fasting blood glucose level. - Stimulates glucose transport in skeletal muscle via the GLUT 4 translocation and phosphorylation of AMPK and Akt.	[121]
	Male Sprague–Dawley rats (CGA (120 mg · kg–1)	- Improved glucose metabolism as seen in decreased AUC.	[134]

Caffeic acid has been studied extensively in experimental diabetes and related complications. Caffeic acid lowered blood glucose level in C57BL/KsJ-*db/db* mice [115] and Streptozotocin (STZ)-induced diabetic rats [116]. Caffeic acid also improved insulin level in plasma of male Balb/cA mice [11] and improved glucose intolerance in high fat diet fed male mice [117]. Caffeic acid improved insulin resistance by promoting insulin receptor tyrosyl phosphorylation, up-regulate the expression of insulin signal associated proteins, including insulin receptor, phosphatidylinositol-3 kinase, glycogen synthase, and glucose transporter-2, increase the uptake of glucose in tumor necrosis factor-α induced insulin resistant mouse liver FL83B cells [118]. Other studies showed that caffeic acid decreased the inflammatory cytokines [119] and reduced the induction of the inflammatory pathway, c-jun-N- terminal kinase, the nuclear factor kappa B, and cyclooxygenase-2 expression [11]. Furthermore, Caffeic acid increased increased phosphorylation of AMPKs and increased glucose uptake and GLUT4 content in L6-GLUT4*myc* cells [120]. Chlorogenic acid also follows the similar mechanism for improving insulin resistance and diabetes. Chlorogenic acid stimulates glucose transport in skeletal muscle via the GLUT 4 translocation and phosphorylation of AMPK and Akt in db/db mice [121].

## Conclusion

**Fig. 3:**
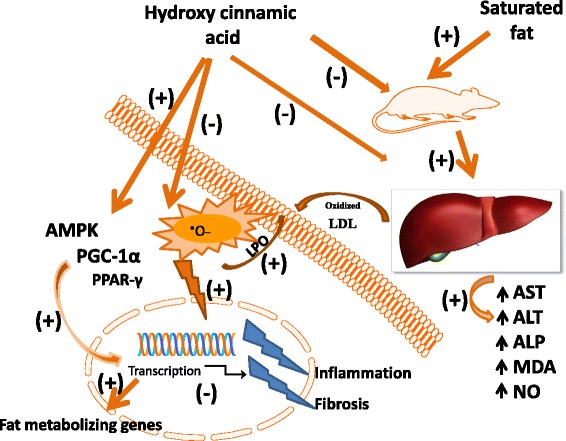
Hypothetical representation of fat metabolism in response to hydroxycinnamic acid derivatives

Recent research has provided the scientific benefit of these phenolic acids and confirmed the important role of phenolic acids in the prevention and treatment of obesity, diabetes and associated disorders. Phenolic acids could favourably affect most of the leading aspects of obesity including diabetes, including insulin resistance, hyperglycemia, hyperlipidemia, and adepocyte dysfunction and inflammation (Fig. 3). Despite the potential benefits of these natural products in preclinical studies, scanty literatures have been found on any beneficial effect from clinical trials of phenolic acids so far. Studies are thus required in humans to confirm the potential benefit of phenolic acids in limiting obesity and other associated disorders. Furthermore, multiple approaches are also needed to overcome limited solubility and poor bioavailability of phenolic acids. These include synthesis of phenolic acids derivatives and development of novel drug delivery system and formulations such as nanoparticles, liposomal encapsulation, emulsions, and sustained released tablets. Therefore, enhanced bioavailability and convinced clinical trial results of phenolic acids could bring these promising natural products to the forefront of therapeutic agents for obesity.

## Abbreviations

AGEs: advanced glycation end products
AMPK: AMP-activated protein kinase
AP-1: activator protein-1 DNA binding
CPT-1: carnitin palmitoyl transferase-1.
EGCG: epigalocatechin gallate
ERK: extracellular signal-regulated protein kinase
GIP: glucose-dependent insulinotropic polypeptide
GLUT: glucose transporter
MAPK: mitogen-activated protein kinases
MMP-2: matrix metalloproteinase-2
NF-κB: nuclear factor kappa-B
Nrf2: nuclear factor erythroid 2 related factor 2
PEPCK: glucose-6-phosphatase and phosphoenolpyruvate carboxykinase
PGI2: increased prostacyclin I2
PI3: phosphoinositide 3 kinase/protein kinase B
PPARα: peroxisome proliferator-activated receptor alpha
ROS: reactive oxygen species
SGLT: sodium-dependent glucose transporter
TGF-β: transforming growth factor-β
TXA2: thromboxane A2
UCP-2: uncoupling protein 2
VEGF: vascular endothelial growth factor
